# Parental Marital Satisfaction and Suicidal Behavior in Preadolescents and Adolescents: The Mediating Role of Positive Youth Development Attributes

**DOI:** 10.3390/ijerph23040468

**Published:** 2026-04-07

**Authors:** Daniel T. L. Shek, Yiting Tang, Xiang Li, Li Zhao

**Affiliations:** 1 Department of Applied Social Sciences, The Hong Kong Polytechnic University, Hong Kong SAR 999077, China; tangyiting.tina@gmail.com (Y.T.); xann.li@polyu.edu.hk (X.L.); 2 Department of Health Policy and Management, West China School of Public Health and West China Fourth Hospital, Sichuan University, Chengdu 610041, China; zhaoli@scu.edu.cn

**Keywords:** marital quality, positive youth development, suicidal behavior, Chinese adolescents, Chinese preadolescents

## Abstract

**Highlights:**

**Public health relevance—How does this work relate to a public health issue?**

- 15.5% of Chinese students aged 9–19 in this study reported at least one form of suicidal behavior, with prevalence reaching 16.4% among preadolescents (aged 9–12), which demands urgent attention.
- Lower parental education was associated with a significantly higher risk of suicidal behavior in children, highlighting socioeconomic disparities in adolescent mental health.

**Public health significance—Why is this work of significance to public health?**

- This study identified positive youth development (PYD) attributes as accounting for the largest proportion of variance across all outcome variables, suggesting they serve as the strongest protective factor against suicidal behavior among those examined.
- Findings revealed marital satisfaction as a protective factor, as it reduced the risk of suicidal behavior in youths indirectly through fostering PYD attributes, with the indirect effect accounting for 56.6% of the total effect.

**Public health implications—What are the key implications or messages for practitioners, policy makers and/or researchers in public health?**

- Suicide prevention strategies should adopt a dual-track approach: strengthening families through marriage education and cultivating holistic psychosocial competencies in adolescents in schools.
- Routine, developmentally sensitive mental health screening should begin in upper primary school (Grades 5–6), and community-based support systems should target families with limited educational resources to reduce structural inequities.

**Abstract:**

**Background**: This study examined the predictive effect of parental marital satisfaction on suicidal behaviors among preadolescents and adolescents in China, with positive youth development (PYD) attributes as a mediator. **Methods**: A total of 3665 matched pairs of students (aged 9–19, 51.3% boys) and their parents completed questionnaires, with parental marital satisfaction reported by parents and suicidal behaviors (ideation, plan, and attempt) and PYD attributes reported by students. **Results**: The prevalence of overall suicidal behavior was 15.5% in this sample, with a higher prevalence observed among those with lower parental education levels. Hierarchical regression and structural equation modeling analyses revealed that: (1) after controlling for socio-demographic variables, parental marital satisfaction negatively predicted suicidal behaviors; (2) PYD attributes negatively predicted suicidal behaviors, accounting for the largest proportion of variance (Δ*R*^2^ range = 0.036–0.102); (3) parental marital satisfaction was positively correlated with PYD attributes; and (4) PYD attributes partially mediated the predictive relationship between parental marital satisfaction and suicidal behavior, with a significant indirect effect (β = −0.06) accounting for 56.6% of the total effect. **Conclusions:** This study illuminates protective pathways through which a positive family environment cultivates individual competencies, ultimately contributing to reduced suicidal behavior.

## 1. Introduction

Suicide among adolescents is becoming increasingly serious. The World Health Organization [1] pointed out that this has become a major public health concern worldwide, as supported by the reports released in both the United States [2] and South Korea [3]. In China, suicide has increased in recent years [4], including among preadolescents aged 9 to 11 [5].

Theoretically, suicide has been widely explained within the framework of “ideation-to-action”, assuming that the development of suicidal thoughts (ideations) and the progression from them to suicidal attempts are distinct processes that require different explanations [6]. Within this framework, various theories have emerged. For instance, the Interpersonal Theory of Suicide ([7]) posits that suicidal ideation stems from a perception of burden and obstruction of belonging. In this scenario, the capability to commit suicide is acquired through painful experiences, and this facilitates the transition from ideation to attempt. Similarly, the Integrated Motivational-Volitional model [8] divides suicide into the motivation stage (the development of suicidal intention) and the volition stage (the progression to actual attempt); and factors such as predicament and defeat become key driving forces. More recently, the Three-Step Theory ([9]) posits that hopelessness combined with pain leads to the emergence of suicidal ideation, while the capacity for suicide prompts individuals to attempt it. Studies showed that individuals with a history of suicidal attempts have a higher level of suicidal capability compared to those with suicidal ideation alone [10], indicating that these suicide-related forms are conceptually distinct.

It is a common approach to distinguish between suicidal ideation, plans, and attempts in suicidal behavior research [11,12,13,14]. The three indicators have also been widely employed in both epidemiological surveillance and clinical risk assessment [15,16]. Suicidal ideation refers to the thoughts about ending one’s own life, while planning involves the formulation of specific methods, and attempt refers to the actual act of self-harm with at least a certain intention of death [12,13]. Each of these forms has the potential to evolve into actual deaths [13]. The research revealed that more than half of adolescents who committed suicide had previously engaged in suicidal behavior [17]. Those who had suicidal thoughts and attempts during their childhood are more likely to repeat such actions in the future [18]. Besides, suicidal behavior in one family member may trigger similar behaviors in others [19], highlighting the familial interconnectedness of this issue.

Negative family factors, such as poor family functioning, were associated with suicidal behaviors in young people [20,21]. However, there are two limitations of the existing scientific literature. First, there are few studies on the relationship between parents’ marital quality and suicidal behavior in their offspring. As an illustrative indication of this scarcity, presented in Table 1, a PsycINFO database search up to February 2026 revealed that with reference to adolescent suicidal behavior, suicidal ideation and suicide in adolescents, studies using “marital quality” consistently yielded fewer publications than those using the problem-focused indicator of “marital conflict.” A similar pattern was also observed in broader searches across Web of Science and Scopus. Second, most of the related studies have been conducted in the West. When the keyword “Chinese” was added to the preceding search, the number of identified studies remained limited (see Table 1).

Family ecological models posit that marital quality has a “spillover effect” on parent–child relationships [22], which would eventually influence children’s well-being. The spillover effect thesis posits that emotions, behaviors, and resources generated in the marital relationship would transfer to parenting practices and the broader family climate [22]. Consequently, a high-quality parental marriage generates emotional resources that “spill over” into warmer, more consistent parenting, which in turn fosters a family environment that satisfies children’s basic psychological needs for security and belonging [23]. The theoretical framework is further illuminated by Family Systems Theory [24]. According to Family Systems Theory, the marital subsystem is the core of family functioning; its quality cascades onto other subsystems, such as parent–child interactions.

High-quality parental marriages can benefit child development through multiple interconnected mechanisms. First, a stable and harmonious marital relationship creates a predictable and emotionally secure family environment, which is conducive to children’s psychosocial development [25]. Such emotional security, in turn, reduces the risk of suicidal ideation and attempts [26]. Second, satisfied couples are more likely to make committed emotional and socioeconomic investments in their children, such as providing quality time, educational resources, and emotional availability [27]. These investments enhance children’s self-esteem, serving as a powerful buffer against suicide-related outcomes [28]. Third, positive marital quality facilitates “concerted cultivation”, which is a parenting style characterized by warm, consistent, and developmentally stimulating practices [29]. This parenting approach actively fosters children’s cognitive and emotional competencies, which protect against suicidal behavior [30]. Fourth, parents in satisfying marriages are better able to provide consistent support, which cultivates children’s resilience and self-efficacy, which are well-established protective factors against suicidal ideation and attempts, and foster children’s positive development [31,32]. Empirical studies consistently supported these pathways, showing that higher parental marital quality was generally associated with better child well-being [33,34].

Besides positive marital quality, personal developmental assets also serve as protective factors for adolescent suicidal behavior. Unlike the deficit view of adolescence that emphasizes problems and pathologies, the positive youth development (PYD) approach focuses on strengths, potentials, and developmental assets [35]. PYD is a research paradigm that encompasses a holistic view of young people, emphasizing the dynamic interplay between individual attributes and supportive environmental factors, such as family, school, and community, which fosters growth and resilience [36]. Several frameworks have been commonly proposed within the PYD literature. Catalano et al. [37] proposed a PYD framework based on an evaluation of PYD programs that is composed of 15 PYD constructs, such as resilience, emotional and cognitive competence, self-determination, and self-efficacy. Another prominent model is the “Five Cs” framework developed by Lerner and colleagues [38], which posits that PYD is characterized by Competence, Confidence, Connection, Character, and Caring, with a sixth C (i.e., Contribution) emerging when all five Cs are sufficiently developed. A related yet distinct framework is social and emotional learning (SEL), which focuses on five core competencies that collectively improve mental health and social behavior [39]. While SEL emphasizes skill acquisition within educational settings, the PYD approach adopts a broader ecological perspective that integrates family, school, and community systems as developmental assets [40].

Studies consistently supported that PYD attributes serve as protective factors against problematic behaviors such as Internet addiction and bullying among Chinese adolescents [41,42]. Regarding the specific link between PYD and suicidal behavior, studies showed that PYD attributes were negatively related to adolescent suicidal behavior [43,44] and they mediated the association between family functioning and adolescent risk behaviors [45]. A recent nested case–control study further found that resilience (a core PYD attribute) reduced the odds of progression from suicidal ideation to plan and from ideation to attempt among Chinese adolescents [46].

In addition, while family and geographical correlates such as residing in rural or mountainous areas [47] and having parents with low educational attainment [48,49] were more consistently identified as risk factors for adolescent suicidal behavior, findings regarding individual-level correlates of suicidal behavior, such as age and gender, remain mixed [50]. Focusing on Chinese societies, some studies suggest that only-children are more vulnerable to the effects of a fragile family environment (e.g., parental conflict, high expectation pressure), thereby increasing their risk of non-suicidal self-injury [51,52]. However, empirical studies directly examining the association between only-child status and adolescent suicidal behavior remain scarce.

### The Present Study

Suicide among children and adolescents is an escalating public health crisis worldwide [1,2,3]. In China, the situation is alarming, and suicide has become the leading cause of death among Chinese youth [4,5,53]. Despite this alarming public health burden, significant gaps remain in the scientific literature.

First, although China has a large population of young people, related research on suicidal behavior is sparse, particularly for preadolescents. Alarmingly, evidence showed that suicidal ideation and attempts are already present among children under 12 years old [5]. Current empirical research remains highly concentrated on adolescent groups aged 12 and above (e.g., [43,44]). Our literature search further highlights this critical research gap. When “preadolescents” was used instead of “adolescents” as the keyword, no publication was found in the search results (see Table 1). This lack of attention to younger children is particularly concerning, given that preadolescence is a critical developmental stage.

Second, although marital quality can be examined through both conflict and satisfaction measures [54], existing research has focused on “problem” indicators such as marital conflict [55]. Although scholars emphasized understanding marital quality through positive concepts such as “satisfaction” [56] or “harmony” [57,58], studies utilizing positive indicators to explore the impact of parents’ marital quality on children’s development are rather scarce [55,59]. According to our PsycINFO search, the research that operationalized marriage quality as “marriage conflict” is almost twice as many as those using “marital satisfaction” measures (see Table 1). This is a relevant real-world concern as the recent large-scale evidence from China shows that poor interparental relationship quality, rather than divorce per se, was associated with elevated suicide risk in adolescents [60], underscoring family-centered intervention, particularly improving marriage quality as a critical protective mechanism.

Third, regarding theoretical model integration, there are few studies that jointly incorporate family protective factors (e.g., parental marital satisfaction) and individual protective factors (e.g., PYD) into a model to explore how they contribute to youth suicidal behavior. This study attempted to investigate the mediating role of PYD in this relationship. Understanding the pathways through which family environment translates into individual-level protection for children can help refine intervention measures.

Fourth, existing empirical studies on the association between PYD attributes and adolescent suicidal behavior are limited by sample size constraints [20,44]. Methodologically, a small sample is prone to unstable estimates.

Finally, existing studies often lacked multi-informant data collection, simultaneously based on parent reports and child reports. Methodologically speaking, employing a multi-informant perspective can yield more comprehensive measurement information and, when appropriately applied, help reduce common method bias associated with a single informant [61]. Furthermore, there are significant validity limitations when children directly report on “parental marital satisfaction” [62]. Given these considerations, a multi-informant design was appropriate for this study. In this study, we invited parents to respond to a measure of marital satisfaction and children to report their PYD qualities and suicidal behaviors.

Against the above background, this study examined five research questions:

***Research Question* 1**: What is the prevalence of suicidal behavior in Chinese preadolescents and adolescents, and how is it related to socio-demographic factors, including age, gender, grade, area (rural versus urban), only-child versus non-only-child status, and parental education levels?

***Research Question* 2**: Is parental marital satisfaction associated with preadolescent and adolescent suicidal behavior? As marital quality may have a spillover effect within the family [22], we hypothesized that higher levels of parental marital satisfaction would predict lower rates of suicidal behaviors in preadolescents and adolescents (**Hypothesis 1**).

***Research Question* 3**: Do PYD attributes predict preadolescent and adolescent suicidal behavior? With reference to previous studies indicating that PYD attributes are negatively related to adolescent suicidal behavior (e.g., [43,44,46]) and other risk behavior (e.g., [41,42]), we predicted that stronger PYD attributes would correspond to reduced suicidal behaviors in preadolescents and adolescents (**Hypothesis 2**).

***Research Question* 4**: Is parental marital satisfaction related to PYD attributes? Previous studies suggested that positive family factors were positively correlated with PYD attributes [63,64]. Besides, as the marital system is a major component of the family system [24], we anticipated that parental marital satisfaction would be positively associated with children’s PYD attributes (**Hypothesis 3**).

***Research Question* 5**: Do PYD attributes mediate the predictive relationship between parental marital satisfaction and suicidal behavior among preadolescents and adolescents? With reference to the mediating role of PYD attributes between family functioning and adolescent developmental outcomes [45], and PYD attributes which represent crucial developmental assets essential for navigating challenges [65], we integrated family ecological model and PYD framework and hypothesized that PYD attributes would mediate the predictive relationship between parental marital satisfaction and suicidal behavior among preadolescents and adolescents (**Hypothesis 4**).

## 2. Materials and Methods

### 2.1. Participants and Procedures

This research drew data from the Chengdu Positive Child Development (CPCD) survey, a multidisciplinary school-based cohort study investigating the antecedents and consequences of psychosocial development among children and adolescents in Sichuan Province, China. Baseline data were collected between December 2019 and January 2020 from 8825 children and adolescents aged 6 to 16 years across five primary and secondary schools (for more details, see [66]).

To better understand the relationships between students’ family environments, lifestyle behaviors, and their physiological and psychological development, an assessment of guardian (parental) marital quality was introduced in the fourth wave (collected in June 2022). The present study is cross-sectional in nature and utilizes data from this fourth wave, which marked the first inclusion of marital quality measures. At Wave 4, the total student sample consisted of 5819 participants.

Prior to the survey, informed consent was obtained from all participating students and their parents. Students completed the Student Questionnaire in the classroom under the guidance of headteachers and trained research staff. Empirical research has established the validity of self-report methods with young children (aged 4–12; e.g., [67]), with children as young as 6 possessing the cognitive capacity to understand and report their emotions, behaviors, and thoughts [68]. Besides, students were encouraged to raise questions at any time if they had any doubts about the questions or items. Research staff would provide instructions and explanations. Each student was also instructed to take the Parent Questionnaire home for completion by one parent. The analytical sample of this study comprises 3665 matched student-parent dyads from Wave 4, representing the subset of participants for whom both student and parent questionnaires were completed. The study was approved by the Institutional Review Board of Sichuan University (Approval No.: K2020025).

### 2.2. Instruments

#### 2.2.1. Parental Marital Satisfaction

The Kansas Marital Satisfaction Scale (KMSS; [69]) was utilized to assess parental marital satisfaction, including 3 items (“*How satisfied are you with your marriage?*”, “*How satisfied are you with your spouse?*”, and “*How satisfied are you with the relationship between you and your spouse?*”). All items are rated using a 7-point Likert scale (1 = “extremely dissatisfied”; 7 = “extremely satisfied”), with higher scores indicating greater marital satisfaction. Prior studies reported the good validity and reliability of the Chinese version of the KMSS. For instance, Shek and Tsang [70] examined this 7-point scale in a Hong Kong sample and reported excellent internal consistency (Cronbach’s α = 0.92), with corrected item-total correlations exceeding 0.83. Li and Chen [71] further established normative data for its Chinese version in both Beijing and Hong Kong samples (total N = 1218) using the same 7-point format, reporting high internal consistency (α = 0.93 for the Beijing sample; α = 0.96 for the Hong Kong sample) and well-established validity. The 7-point version has also been widely used in recent studies with Chinese samples (e.g., [72,73,74]). In this study, its internal consistency was assessed using both Cronbach’s α and McDonald’s ω. The results indicated excellent reliability (α = 0.97, ω = 0.97). Factor loadings ranged from 0.97 to 0.98, supporting the unidimensional structure of the scale.

#### 2.2.2. Positive Youth Development

Students’ positive qualities were measured via the 80-item version of the Chinese Positive Youth Development Scale (CPYDS; [75]), which is divided into 15 dimensions (e.g., emotional competence, self-efficacy, resilience, spirituality, etc.). The items in the spirituality dimension are rated on a 7-point Likert scale (1 = “most negative”; 7 = “most positive”), while the other dimensions are rated on a 6-point Likert scale (1 = “strongly disagree”; 6 = “strongly agree”). Higher scores reflect higher levels of PYD. Past research has demonstrated a good fit for its 15-factor structure (CFI = 0.98, NNFI = 0.98, RMSEA = 0.06, SRMR = 0.05) [75]. Multigroup confirmatory factor analyses demonstrated that this factor structure was invariant across gender in terms of configural invariance (CFI = 0.98, NNFI = 0.98, RMSEA = 0.06, SRMR = 0.05) [75]. The scale has been widely used in previous studies with Chinese children and adolescents [76,77,78], with consistently reported high internal consistency (Cronbach’s α ranged from 0.95 to 0.98). In the present study, McDonald’s ω values ranged from 0.76 (Self-efficacy) to 0.95 (Spirituality) across the 15 subscales, and all subscales had Cronbach’s α ≥ 0.75. The total scale demonstrated excellent reliability (ω = 0.98, α = 0.97). In this study, it was administered in a classroom setting with brief breaks to minimize fatigue. Besides, past studies have also supported the acceptability of the full 80-item length of this scale in the youth population [76,77,78].

#### 2.2.3. Adolescent Suicidal Behavior

Three items adapted from Shek and Yu [79] were used to assess the three indicators of suicidal behaviors, including suicidal ideation (“*Have you ever contemplated seriously about attempting suicide?*”), suicide plan (“*Have you formulated a detailed plan on how you would carry out a suicide attempt?*”), and suicide attempt (“*How many times have you actually attempted suicide?*”). For each item, students were asked to report whether they had engaged in the behavior in the past year via a 4-point Likert scale (1 = “never,” 2 = “once,” 3 = “twice,” 4 = “three times or more”). This three-item measure has been widely used in previous studies with Chinese children and adolescents [79,80,81], demonstrating acceptable reliability and validity (Cronbach’s α = 0.68; [79]). According to Wei and Zhang [82], brief measures (including single-item measures) are appropriate when the following conditions are met: (a) large sample size, (b) the construct is unidimensional, (c) the construct is specific and clearly defined, and (d) time constraints exist. The present study satisfied all these criteria. Moreover, using a single-item reduced respondent burden and potential discomfort when answering sensitive questions about suicide [12,16]. The three-item measure also showed excellent internal consistency in the present study, supported by both Cronbach’s α (0.93) and McDonald’s ω (0.94) values. Factor loadings ranged from 0.85 to 0.96, suggesting good convergent validity among the three items. In this study, both the individual item scores and the average score across the three items were used. The average score was used as an indicator reflecting overall suicidal tendency, consistent with prior research [79]. The individual item scores were employed to capture the distinctiveness of each suicidal behavior form.

### 2.3. Data Analysis

Statistical analyses were performed using SPSS 27.0 [83] and Mplus (version 8.11; [84]). To address Research Question 1, students’ responses on the suicidal behavior measures were dichotomized for descriptive comparisons. For each of the three individual items, responses of “1” (“never”) were classified as the “low-risk group” (i.e., no history of that behavior), whereas others were classified as the “high-risk group” (i.e., at least one instance of the behavior). Similarly, the average score across the three items was dichotomized using the same criterion to facilitate descriptive analyses.

Additionally, to better understand the distribution of the prevalence across developmental stages and examine differences, students were categorized into four groups based on cutoffs of developmental stages established in prior research [85,86]: “preadolescence” (aged 9–12), “early adolescence” (aged 13–14), “middle adolescence” (aged 15–16), and “late adolescence” (aged 17–19). Chi-square tests examined differences in the prevalence of suicidal behaviors across socio-demographic characteristics. When overall chi-square tests were significant, a post hoc comparison would be conducted using the standardized residuals with Bonferroni correction to identify the specific categories that show significant differences.

To answer Research Questions 2 and 3, hierarchical multiple regression analyses examined whether parental marital satisfaction and PYD attributes predicted suicide-related behaviors (overall suicidal behavior, suicidal ideation, suicide plan, suicide attempt), after accounting for socio-demographic variables. For dependent variables in these analyses, the three individual item scores were used separately to capture the distinctiveness of each form, while the average score across the three items was used as an indicator of the tendency towards overall suicidal behavior. Besides, the approach of using the original continuous scores in the primary inferential analyses allows for preserving statistical power. Each regression model consisted of three sequential blocks of predictors: Block 1 entered socio-demographic variables (age, gender, grade, only-child status, residential area, parental education level) as covariates, with parental marital satisfaction added in Block 2, and the total PYD score added in Block 3. The change in *R*^2^ for each block was examined.

The selection of covariates was guided by prior literature identifying these factors as relevant correlates of adolescent suicidal behavior [47,48,49,50,51,52]. Besides, developmental stage groups were not included in the models, given their conceptual overlap and high correlation with grade. Grade alone was retained as a more parsimonious indicator of school level. Given that the sample size (N = 3665) substantially exceeded the number of predictors, the risk of overfitting was considered to be low.

To address Research Question 4, Pearson correlation analyses examined the relationships among parental marital satisfaction, PYD attributes, and suicidal behaviors. To address Research Question 5, structural equation modeling (SEM) was performed to examine the hypothesized mediation model for two main reasons. First, it allows PYD attributes to be modeled as a latent variable using its 15 dimensions, thereby reducing measurement error and yielding more precise parameter estimates [87]. Second, it enables simultaneous estimation of the full mediation model with all indicators, offering a more rigorous framework for testing indirect effects [88]. In the mediation model, parental marital satisfaction was specified as an observed exogenous variable. PYD attributes were modeled as a latent variable with its 15 dimensions serving as observed indicators, and suicidal behavior as a latent variable with the three behavioral indicators (ideation, plan, attempt). Consistent with multiple regression analyses, the original continuous scores of suicidal behavior were used in the SEM to preserve statistical power. Gender and grade were included as covariates. The significance of the indirect effect was tested using the bias-corrected nonparametric percentile Bootstrap method with 1000 resamples and the Maximum Likelihood (ML) estimator. Model fit was evaluated using the root mean square error of approximation (RMSEA), comparative fit index (CFI), Tucker–Lewis index (TLI), and standardized root mean square residual (SRMR). Acceptable fit was defined as RMSEA ≤ 0.08, SRMR ≤ 0.08, and CFI/TLI ≥ 0.90, with CFI ≥ 0.95 indicating good fit [89].

## 3. Results

### 3.1. Suicidal Behaviors and Socio-Demographic Correlates

Among the 3665 students, the prevalence of overall suicidal behavior (i.e., having experienced at least one form of suicidal behavior) was 15.5% (n = 569). For specific indicators, the prevalence rates were 13.9% (n = 509) for suicidal ideation, 8.1% (n = 296) for suicide plan, and 6.4% (n = 234) for suicide attempt.

The sample comprised 1881 (51.3%) boys and 1784 (48.7%) girls. These students, aged 9 to 19 (M = 12.59, SD = 1.65), were classified into four developmental stage groups: preadolescence (aged 9–12; n = 1927, 52.6%), early adolescence (aged 13–14; n = 1182, 32.3%), middle adolescence (aged 15–16; n = 542, 14.8%), and late adolescence (aged 17–19; n = 11, 0.3%). Regarding grade, the sample distribution was: Grade 4 (n = 399, 10.9%), Grade 5 (n = 911, 24.9%), Grade 6 (n = 917, 25.0%), Grade 7 (n = 425, 11.6%), Grade 8 (n = 685, 18.7%), and Grade 9 (n = 328, 8.9%). For the residential area, 2495 (68.1%) students were from urban areas and 1170 (31.9%) from rural areas. Regarding only-child status, 1226 students (33.5%) were only-children, and 2439 (66.5%) were non-only-children. Parental education levels were distributed as follows: primary school (n = 312, 8.5%), middle school (n = 1417, 38.7%), high school (n = 1175, 32.1%), and Bachelor’s degree or higher (n = 761, 20.8%).

Chi-square tests were conducted to examine differences in the prevalence of suicidal behaviors across socio-demographic characteristics. The results are summarized in Table 2. Most socio-demographic factors revealed no statistically significant associations with suicidal behaviors, including developmental stage, grade, gender, residential area, and only-child status.

However, parental education level showed significant associations with suicidal behaviors. The prevalence of overall suicidal behavior, suicidal ideation, and suicide plan differed significantly across parental education categories (overall suicidal behavior: χ^2^ (3, *N* = 3665) = 13.74, *p* = 0.003, Cramér’s V = 0.06, 95% CI [0.04, 0.10]; suicidal ideation: χ^2^ (3, *N* = 3665) = 14.91, *p* = 0.002, Cramér’s V = 0.07, 95% CI [0.04, 0.11]; suicide plan: χ^2^ (3, *N* = 3665) = 10.19, *p* = 0.017, Cramér’s V = 0.05, 95% CI [0.03, 0.09]). Post hoc comparisons indicated that students whose parents had a primary or middle school education exhibited higher prevalence rates compared to those whose parents held a Bachelor’s degree or higher. However, the effect sizes were small, suggesting modest practical significance.

### 3.2. Correlations Between Parental Marital Satisfaction, PYD Attributes, and Suicidal Behaviors

Hierarchical multiple regression results are presented in Table 3, which displays the key predictors (i.e., parental marital satisfaction and total PYD) along with their coefficients and model fit statistics. The control variables (age, gender, grade, only-child status, residential area, and parental education level) were included in all blocks and are listed in the table footnote. Full regression results, including coefficients for all control variables across three blocks, are available as Supplementary Materials.

After controlling for socio-demographic variables, parental marital satisfaction negatively predicted all forms of suicidal behavior (Δ*R*^2^ range = 0.004–0.008; *p*s < 0.01). Although statistically significant, these Δ*R*^2^ values were small in magnitude. The subsequent inclusion of total PYD score also yielded significant negative predictions for all outcomes (Δ*R*^2^ range = 0.036–0.102; *p*s < 0.001), which were also modest in magnitude but consistently larger than those contributed by parental marital satisfaction. These findings support Hypotheses 1 and 2. In all final models, total PYD score accounted for the largest proportion of variance explained (β range = −0.33 to −0.20; *p*s < 0.001). Notably, with PYD added, the predictive effect of parental marital satisfaction became non-significant (see Table 3), indicating that the association between parental marital satisfaction and adolescent suicidal behaviors may be largely explained by PYD attributes. Moreover, grade remained a significant predictor in the final models. Compared to students in Grade 9 (the reference group), those in Grades 5 and 6 reported significantly higher levels of overall suicidal behavior, suicidal ideation, and suicide plan (β range = 0.10–0.16, *p*s < 0.05; see Supplementary Table S1).

Moreover, variance inflation factors (VIFs) were computed for all predictors to assess multicollinearity. The VIFs (see Table 3) for parental marital satisfaction (1.02–1.06 across models) and total PYD (VIF = 1.08) were well below the commonly recommended threshold of 5 [90], indicating that multicollinearity did not affect the stability of these estimates. Some control variables, particularly grade-level dummy variables and age, showed higher VIFs (ranging from 8.59 to 9.85; see Supplementary Table S1), which is expected given the natural correlation between age and grade in school-based samples [91]; however, these were control variables rather than focal predictors.

Table 4 presents mean values, standard deviations, and correlation coefficients between suicidal behaviors, PYD attributes, and parental marital satisfaction. Parental marital satisfaction was negatively correlated to all forms of suicidal behaviors (*p*s < 0.001). PYD attributes were negatively associated with all suicidal behavior indicators (*p*s < 0.001). Furthermore, parental marital satisfaction was positively correlated to all dimensions of PYD (*p*s < 0.001), thus supporting Hypothesis 3.

### 3.3. The Mediating Role of PYD Attributes

Prior to testing the mediation model, we examined whether multicollinearity among 15 PYD attributes would affect the stability of the estimates by computing VIFs when the 15 indicators entered as separate predictors in auxiliary regression models. The VIF values ranged from 1.74 to 3.88, all below the commonly accepted threshold of 5, indicating that multicollinearity did not distort the coefficient estimates.

**Figure 1:**
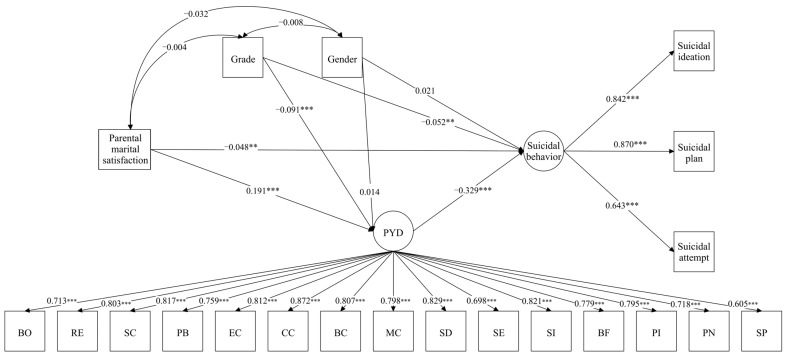
The mediating effect model coefficient of parental marital satisfaction on adolescent suicidal behaviors. BO: Bonding; RE: Resilience; SC: Social competence; PB: Recognition for positive behavior; EC: Emotional competence; CC: Cognitive competence; BC: Behavioral competence; MC: Moral competence; SD: Self-determination; SE: Self-efficacy; SI: Clear and positive identity; BF: Beliefs in the future; PI: Prosocial involvement; PN: Prosocial norms; SP: Spirituality. Single−headed arrows represent standardized path coefficients. Double−headed arrows indicate covariances among the exogenous variables. The data in the figure are standardized coefficients, ** *p* < 0.01, *** *p* < 0.001.

The hypothesized structural equation model demonstrated acceptable fit to the data: χ^2^ (182) = 2831.22, *p* < 0.001, CFI = 0.90, TLI = 0.89, RMSEA = 0.06 (90% CI: 0.06–0.07), SRMR = 0.05. Standardized path coefficients are illustrated in Figure 1. Parental marital satisfaction exerted a significant positive effect on PYD attributes (β = 0.19, *p* < 0.001), which in turn negatively predicted suicidal behaviors (β = −0.33, *p* < 0.001). The direct effect of parental marital satisfaction on suicidal behaviors also remained significant (β = −0.05, *p* < 0.01).

Mediation analysis using bias-corrected bootstrap (1000 resamples) revealed a significant indirect effect of parental marital satisfaction on suicidal behaviors through PYD attributes (β = −0.06, *p* < 0.001; see Table 5). The 95% confidence intervals (CI) for this indirect path (95% CI: −0.08, −0.04) did not contain zero, confirming a significant mediating effect. The indirect effect accounted for 56.6% of the total effect (standardized total effect = −0.11, *p* < 0.001). These results confirm that PYD attributes partially mediated the relationship between parental marital satisfaction and suicidal behaviors, supporting Hypothesis 4. Notably, while the 56.6% figure of indirect effects is considerable, due to the modest direct effect (β = −0.05) and the cross-sectional nature of the data, this proportion should not be overly interpreted as support for the causal mechanism.

## 4. Discussion

This study examined the relationships between parental marital satisfaction, PYD attributes, and suicidal behaviors among preadolescents and adolescents within a Chinese context. The prevalence data demand urgent attention. Overall, 15.5% of students in this study reported at least one form of suicidal behavior in the past year, with 13.9% for suicidal ideation, 8.1% for suicide plans, and 6.4% for suicide attempts. These figures are consistent with national meta-analytic estimates [4]. Additionally, a recent survey also warned of a worrying backdrop of rising youth suicide mortality in China since 2017 [53]. Although our cross-sectional data cannot reflect the trends over time, the high prevalence we observed still echoes the warning from mortality rates. Together, these findings underscore the urgent need for proactive prevention and intervention among Chinese youth today.

Alarmingly, across Grades 4 to 6, the prevalence of suicidal behavior ranged from 14.9% to 17.0%, with a comparable rate of 16.4% among preadolescents (aged 9–12 years). Grades 5 and 6 represent a critical developmental transition, during which children face various pressures, including academic demands, peer reorganization, identity formation, and pubertal onset, while cognitive and emotional regulation capacities remain under development. This combination may create a window of heightened vulnerability, thereby increasing the risk of internalizing problems. Therefore, we urge earlier mental health screening for Chinese school-aged children, and recommend that preventive education and support systems extend to younger age groups.

Contrary to some previous findings [47], no significant differences were observed across gender, residential area, or only-child status. One possibility is that contemporary stressors (e.g., academic pressure, digital media exposure) may affect adolescents across different demographic backgrounds, though this interpretation remains speculative and warrants further investigation. Consistent with previous research [48,49], lower parental education was associated with a higher risk of suicidal behavior in children. Parents with higher education may possess more parenting knowledge, emotion regulation skills, and economic resources [49], which could enable them to provide more stable environments and effective support for their children. Given that family counseling and interventions remain limited in China, strengthening community-based support systems (e.g., parent education, support groups) could be a direction to enhance protective resources for disadvantaged families.

For Research Question 2, parental marital satisfaction negatively predicted all forms of suicidal behavior, after controlling for socio-demographic variables, thus corroborating Hypothesis 1. However, the effect sizes for these direct associations were small (Δ*R*^2^ ranging from 0.004 to 0.008), indicating that marital satisfaction alone explains a limited proportion of variance in adolescent suicidal behavior. This is not a surprise because marital satisfaction and suicidal behavior were reported by different informants. This finding supports the protective value of positive family characteristics indexed by parental marital satisfaction for the healthy development of children, complementing the extensive literature focusing on family risk factors such as conflict, divorce, and dysfunction. By shifting the perspective toward protective factors, this study underscores the importance of positive marital quality as a key family-level variable influencing youth development, aligning with research emphasizing the protective effects of family harmony [25,27,29,33] and family dynamics [46] on adaptive developmental trajectories for youths.

The present findings align with Family Systems Theory, particularly those emphasizing the spillover effect. The marital subsystem constitutes the core of family functioning, and its quality influences other subsystems. Emotional resources generated in a satisfying marriage may transfer to warmer, more consistent parenting practices, which in turn foster a family environment conducive to children’s psychosocial development. The observed association between parental marital satisfaction and reduced suicidal behavior in this study is consistent with this theoretical chain, suggesting that a harmonious marital relationship may serve as a foundational resource for healthy family functioning [22,29]. In the Chinese cultural context, the protective role of a high marital quality may carry additional significance. Traditional Chinese culture places a high value on family harmony, reflected in the concept that “family harmony leads to prosperity” [92]. Hence, in the Chinese cultural context, harmonious marriages may carry additional symbolic weight, presenting cultural expectations and family unity. This reinforces their protective function for children’s development. Studies showed that Chinese parents’ marital quality was closely linked to children’s developmental outcomes, with family harmony serving as a crucial mediator [54]. The strong emphasis on filial piety and collective family well-being in Chinese societies may amplify the spillover effects of marital quality onto children’s functioning [93].

For Research Question 3, PYD attributes accounted for the largest proportion of variance in all suicidal behavior outcomes among the measures examined, supporting Hypothesis 2. This finding is consistent with the PYD theoretical approach that emphasizes strengths, potentials, and developmental assets [35,36], which moves beyond a deficit-focused perspective. PYD attributes, such as emotional competence, resilience, self-efficacy, and beliefs in the future, may function as an internal psychological resource reservoir, potentially equipping young people to cope adaptively with stress, maintain hope during adversity, and actively seek social support [43,94,95,96,97]. Our findings align with other research based on the same dataset (e.g., [64]), while also supporting the broader literature (e.g., [43,44,46]). Taken together, these findings suggest that PYD attributes represent a potentially important protective factor against youth risk behaviors.

Notably, when PYD was entered into the model, the direct effect of parental marital satisfaction became non-significant. This pattern is consistent with the hypothesis that youth psychosocial competencies may play a mediating role in the family-suicidality link. The finding also responds to recent calls for identifying developmental strengths that can buffer against adolescent suicidal behavior [50]. The finding that PYD largely accounted for the association between marital quality and suicidal behavior suggests that one pathway through which positive family contexts confer protection is by nurturing these developmental assets.

These findings have potential implications for prevention and intervention. Integrating PYD principles into school and family education may be a promising strategy. This is particularly relevant in the Chinese context, where educational systems have historically prioritized academic achievement over holistic psychosocial development. Evidence-based school programs offer a solution. For instance, a large-scale, curriculum-based intervention project implemented across Hong Kong and the Chinese Mainland, “Positive Adolescent Training through Holistic Social Programs” (P.A.T.H.S.), has demonstrated that PYD attributes could be systematically cultivated through well-structured curricula with reported reductions in problem behaviors and enhancements in well-being among youths [96,97,98]. Similarly, another PYD-based intervention programme in the Chinese Mainland has also shown positive effects on improving key PYD attributes such as resilience, self-efficacy, and social competence, as well as enhancing well-being among elementary school students who are migrant children [99]. These examples illustrate that structured PYD interventions are feasible in Chinese school settings and may help translate the protective mechanisms identified in this study into practice. Scaling such PYD-oriented approaches across Chinese schools could meet the current urgent need for strengthening prevention.

In terms of Research Questions 4 and 5, our findings suggest that parental marital satisfaction was positively associated with all 15 dimensions of PYD attributes, and PYD partially mediated the relationship between marital satisfaction and suicidal behavior, with the indirect effect accounting for 56.6% of the total effect, supporting Hypotheses 3 and 4. This mediation model combines Family Systems Theory with the spillover effect model, suggesting that harmonious marriages serve as interpersonal resources (e.g., partner support, positive emotions) for parents, which facilitates the spillover effect into supportive parenting. A positive family climate emerges from this process and enhances family adaptive capacity, in turn, helps children develop their own psychological resources. These individual resources (PYD attributes) then become the proximal mechanisms that directly protect against risk behaviors. However, given the cross-sectional nature of the data, the proportion of the total effect mediated (56.6%) should be interpreted as a statistical description of the indirect pathway within the current sample rather than a precise estimate of causal contribution. The modest direct effect (β = −0.05) further suggests that the overall association between parental marital satisfaction and suicidal behavior is small, regardless of the proportion mediated.

Our findings also align with recent research trends. Scholars increasingly emphasized the need to investigate multi-level protective factors. For example, Ortiz-Sánchez et al. [100] reported that family cohesion played a central role in protecting against adolescent psychological crises. Building on this line of inquiry, this study extends this work by showing that marital relationship quality at the family subsystem level translates into individual protection through fostering children’s PYD attributes. This specification of the mechanism offers clearer targets for intervention design.

### 4.1. Theoretical and Practical Implications

This study carries both theoretical and practical implications. Theoretically, this study advances an integrated model on the link between family resources and individual psychosocial resources and reduction in risk behavior that synthesizes Family Systems Theory (those emphasizing spillover effect), and PYD frameworks. It delineates a specific mechanism by which family environment influences youth outcomes through cultivating developmental assets.

Practically, the findings suggest three interconnected strategies. First, family-focused interventions that introduce marriage education or relationship enhancement programs for parents within the community system in China would be helpful. Given declining marital satisfaction in China [101], evidence-based marriage enrichment programs [102] that aim to improve spousal communication quality and conflict resolution skills could indirectly benefit children by improving a healthier family ecological environment. Recent experimental studies support the feasibility and effectiveness of such approaches in Chinese contexts. For example, empirical evidence supported that a marital communication program could effectively improve marital satisfaction among Chinese couples [103]. The second is youth-focused interventions. The protective effect of PYD attributes advocates for prioritizing systematic psychosocial competencies cultivation in Chinese school-aged children within educational systems and social services. The third is developmentally sensitive screening. The elevated risk in Grades 5–6 argues for establishing routine and universal mental health and risk behavior screening mechanisms starting from upper primary school, with stage-appropriate tools.

### 4.2. Study Limitations

Despite its pioneer nature in the Chinese context, this study has several limitations. First, as cross-sectional data preclude causal inference, longitudinal designs are needed in future research to establish temporal order and test mediation stability. Second, the data were drawn from several schools in Chengdu, Sichuan Province, which limits generalizability to other regions in China. More replications across diverse Chinese young populations are needed. Third, reliance on self-report may introduce bias despite the multi-informant design being utilized. Future studies could consider incorporating multi-modal data, such as behavioral observations, interviews, and physiological indicators. Fourth, only one parent per family completed the questionnaire, and the specific parent (mother vs. father) was not identified. Given that couples may differ in their perceptions of marital quality and their influence on children, this lack of specification may introduce systematic bias. Future research should consider obtaining data from both parents when possible to allow for a more comprehensive assessment of family dynamics. Nevertheless, it is noteworthy that this is a common approach to invite one parent in a family to participate in similar studies. Finally, the effect sizes of the significant findings related to marital quality were modest. While this is not unreasonable because multiple informants were recruited in this study [104], it also suggests that other unmeasured factors should be included in future studies.

## 5. Conclusions

Focusing on the Chinese context and utilizing multi-informant data, this study provided support for the protective pathway in which parental marital satisfaction influences adolescent suicidal behavior through PYD attributes. The main findings confirm that higher parental marital satisfaction directly predicts lower risk of suicidal behavior and that adolescent PYD attributes partially mediate this relationship. This mediation pathway of “parental marital satisfaction (positive family resource) → PYD attributes (individual psychosocial resource) → reduced risk behavior” provides an empirical model for understanding the synergistic action of multi-level protective factors. The findings advocate for an integrated intervention approach for suicide prevention combining promotion of family competence and adolescent competence through systematic cultivation of psychosocial competencies among youths, with marriage education and family support services, thereby optimizing the micro-system environment for youth development.

## Figures and Tables

**Table 1:** Number of studies on marital quality and adolescent suicidal behavior captured by PsycInfo (in February 2026).

Search Terms	Number of Citations in PsycInfo	Number of Citations in PsycInfo with “Chinese” Being Added as a Search Term
marital quality AND suicidal behavior AND adolescent	14	3
marital quality AND suicidal behavior AND preadolescent	0	0
marital quality AND suicidal ideation AND adolescent	10	3
marital quality AND suicidal ideation AND preadolescent	0	0
marital quality AND suicide AND adolescent	24	4
marital quality AND suicide AND preadolescent	0	0
marital conflict AND suicidal behavior AND adolescent	26	3
marital conflict AND suicidal behavior AND preadolescent	0	0
marital conflict AND suicidal ideation AND adolescent	16	2
marital conflict AND suicidal ideation AND preadolescent	0	0
marital conflict AND suicide AND adolescent	35	5
marital conflict AND suicide AND preadolescent	0	0
marital satisfaction AND suicidal behavior AND adolescent	11	2
marital satisfaction AND suicidal behavior AND preadolescent	0	0
marital satisfaction AND suicidal ideation AND adolescent	8	1
marital satisfaction AND suicidal ideation AND preadolescent	0	0
marital satisfaction AND suicide AND adolescent	16	1
marital satisfaction AND suicide AND preadolescent	0	0

**Table 2:** Descriptive statistics and the detection rate of suicidal behaviors (N = 3665).

Socio-Demographic Category	Overall Suicidal Behavior			Suicidal Ideation			Suicide Plan			Suicide Attempt		
	Low Risk ^§^ (%)	High Risk ^†^ (%)	*p*	Low Risk ^§^ (%)	High Risk ^†^ (%)	*p*	Low Risk ^§^ (%)	High Risk ^†^ (%)	*p*	Low Risk ^§^ (%)	High Risk ^†^ (%)	*p*
Total sample	3086 (84.2)	569 (15.5)	-	3148 (85.9)	509 (13.9)	-	3359 (91.7)	296 (8.1)	-	3422 (93.4)	234 (6.4)	-
Developmental stage			0.140			0.156			0.550			0.509
- Preadolescence	1605 (83.6)	315 (16.4)		1645 (85.6)	276 (14.4)		1752 (91.3)	167 (8.7)		1787 (93.0)	134 (7.0)	
- Early adolescence	1019 (86.4)	160 (13.6)		1035 (87.7)	145 (12.3)		1097 (93.0)	83 (7.0)		1113 (94.4)	66 (5.6)	
- Middle adolescence	452 (83.4)	90 (16.6)		458 (84.5)	84 (15.5)		497 (91.7)	45 (8.3)		508 (93.7)	34 (6.3)	
- Late adolescence	8 (72.7)	3 (27.3)		8 (72.7)	3 (27.3)		10 (90.9)	1 (9.1)		11 (100.0)	0	
Grade			0.077			0.065			0.251			0.387
- Grade 4	337 (85.1)	59 (14.9)		347 (87.4)	50 (12.6)		364 (92.4)	30 (7.6)		370 (93.4)	26 (6.6)	
- Grade 5	755 (83.0)	155 (17.0)		774 (85.1)	136 (14.9)		828 (90.9)	83 (9.1)		843 (92.5)	68 (7.5)	
- Grade 6	761 (83.4)	152 (16.6)		776 (84.9)	138 (15.1)		832 (91.0)	82 (9.0)		853 (93.4)	60 (6.6)	
- Grade 7	377 (88.7)	48 (11.3)		385 (90.6)	40 (9.4)		401 (94.4)	24 (5.6)		406 (95.5)	19 (4.5)	
- Grade 8	585 (85.7)	98 (14.3)		589 (86.2)	94 (13.8)		634 (92.8)	49 (7.2)		639 (93.6)	44 (6.4)	
- Grade 9	271 (82.6)	57 (17.4)		277 (84.5)	51 (15.5)		300 (91.5)	28 (8.5)		311 (94.8)	17 (5.2)	
Gender			0.480			0.438			0.095			0.684
- Boys	1590 (84.8)	284 (15.2)		1623 (86.5)	253 (13.5)		1736 (92.6)	138 (7.4)		1758 (93.8)	117 (6.2)	
- Girls	1496 (84.0)	285 (16.0)		1525 (85.6)	256 (14.4)		1623 (91.1)	158 (8.9)		1664 (93.4)	117 (6.6)	
Child in family			0.556			0.835			0.777			0.581
- Only child	1037 (84.9)	184 (15.1)		1049 (85.9)	172 (14.1)		1119 (91.7)	101 (8.3)		1139 (93.3)	82 (6.7)	
- Non-only child	2049 (84.2)	385 (15.8)		2099 (86.2)	337 (13.8)		2240 (92.0)	195 (8.0)		2283 (93.8)	152 (6.2)	
Region			0.613			0.650			0.341			0.792
- Urban	2105 (84.6)	382 (15.4)		2147 (86.3)	342 (13.7)		2292 (92.2)	194 (7.8)		2326 (93.5)	161 (6.5)	
- Rural	981 (84.0)	187 (16.0)		1001 (85.7)	167 (14.3)		1067 (91.3)	102 (8.7)		1096 (93.8)	73 (6.2)	
Parental education			**0** **.003**			**0** **.002**			**0** **.017**			0.739
- Primary school	249 (79.8) ^a^	63 (20.2) ^a^		252 (80.8) ^a^	60 (19.2) ^a^		278 (89.4) ^a^	33 (10.6) ^a^		291 (93.3)	21 (6.7)	
- Middle school	1177 (83.2) ^a^	238 (16.8) ^a^		1207 (85.2) ^a^	209 (14.8) ^a^		1285 (90.8) ^a^	130 (9.2) ^a^		1318 (93.1)	97 (6.9)	
- High school	995 (85.0) ^a,b^	176 (15.0) ^a,b^		1012 (86.4) ^a,b^	159 (13.6) ^a,b^		1083 (92.5) ^a,b^	88 (7.5) ^a,b^		1099 (93.8)	73 (6.2)	
- Bachelor’s degree/above	665 (87.8) ^b^	92 (12.2) ^b^		677 (89.3) ^b^	81 (10.7) ^b^		713 (94.1) ^b^	45 (5.9) ^b^		714 (94.3)	43 (5.7)	

Note. ^§^ Low risk group: students reporting no history of any form of suicidal behavior. ^†^ High risk group: students reporting at least one instance of suicidal behavior. Developmental stage groups were defined as follows: “preadolescence” (aged 9–12), “early adolescence” (aged 13–14), “middle adolescence” (aged 15–16), and “late adolescence” (aged 17–19). Chi-square tests were conducted to examine differences in the prevalence of suicide-related behaviors across demographic characteristics. Bolded *p*-values indicate statistically significant differences. Superscript letters (e.g., a, b) denote the results of post hoc comparisons using standardized residuals; within the same column, different superscript letters indicate significant between-group differences (*p* < 0.05).

**Table 3:** Model summaries and regression coefficients of parental marital satisfaction and PYD (N = 3665).

Model	Predictors	Dependent Variable																			
		Overall Suicidal Behavior					Suicide Ideation					Suicide Plan					Suicide Attempt				
Block 2		R	R^2^	∆R^2^	F		R	R^2^	∆R^2^	F		R	R^2^	∆R^2^	F		R	R^2^	∆R^2^	F	
		0.12	0.015	0.008 ***	4.13 ***		0.13	0.017	0.008 ***	4.70 ***		0.11	0.012	0.006 ***	3.33 ***		0.08	0.006	0.004 **	1.57 *	
		B	S.E.	β	t	VIF	B	S.E.	β	t	VIF	B	S.E.	β	t	VIF	B	S.E.	β	t	VIF
	1	−0.03	0.01	−0.09	−5.23 ***	1.02	−0.04	0.01	−0.09	−5.17 ***	1.02	−0.03	0.01	−0.08	−4.56 ***	1.02	−0.02	0.01	−0.06	−3.46 **	1.02
Block 3		R	R^2^	∆R^2^	F		R	R^2^	∆R^2^	F		R	R^2^	∆R^2^	F		R	R^2^	∆R^2^	F	
		0.33	0.111	0.096 ***	31.92 ***		0.35	0.119	0.102 ***	34.71 ***		0.29	0.087	0.075 ***	24.35 ***		0.21	0.042	0.036 ***	11.11 ***	
		B	S.E.	β	t	VIF	B	S.E.	β	t	VIF	B	S.E.	β	t	VIF	B	S.E.	β	t	VIF
	1	−0.01	0.01	−0.03	−1.72	1.06	−0.01	0.01	−0.03	−1.54	1.06	−0.01	0.01	−0.02	−1.44	1.06	−0.01	0.01	−0.02	−1.29	1.06
	2	−0.20	0.01	−0.32	−19.69 ***	1.08	−0.30	0.01	−0.33	−20.44 ***	1.08	−0.19	0.01	−0.28	−17.15 ***	1.08	−0.12	0.01	−0.20	−11.59 ***	1.08

Note. 1. Parental marital satisfaction; 2. Total PYD (Positive Youth Development). Control variables (age, gender, grade, only-child status, residential area, parental education level) were included in all blocks; their coefficients are available in Supplementary Materials. * *p* < 0.05; ** *p* < 0.01; *** *p* < 0.001.

**Table 4:** The mean values, standard deviations, and correlation coefficients of suicidal behaviors, parental marital satisfaction, and PYD attributes (N = 3665).

V	M	SD	1	2	3	4	5	6	7	8	9	10	11	12	13	14	15	16	17	18	19	20
1	1.15	0.44	-	-	-	-	-	-	-	-	-	-	-	-	-	-	-	-	-	-	-	-
2	1.22	0.63	0.91 ***	-	-	-	-	-	-	-	-	-	-	-	-	-	-	-	-	-	-	-
3	1.12	0.47	0.89 ***	0.73 ***	-	-	-	-	-	-	-	-	-	-	-	-	-	-	-	-	-	-
4	1.10	0.42	0.78 ***	0.53 ***	0.57 ***	-	-	-	-	-	-	-	-	-	-	-	-	-	-	-	-	-
5	5.58	1.36	−0.09 ***	−0.09 ***	−0.08 ***	−0.06 ***	-	-	-	-	-	-	-	-	-	-	-	-	-	-	-	-
6	5.34	0.81	−0.28 ***	−0.28 ***	−0.24 ***	−0.18 ***	0.17 ***	-	-	-	-	-	-	-	-	-	-	-	-	-	-	-
7	5.43	0.74	−0.3 0***	−0.30 ***	−0.26 ***	−0.18 ***	0.15 ***	0.71 ***	-	-	-	-	-	-	-	-	-	-	-	-	-	-
8	5.05	0.85	−0.17 ***	−0.18 ***	−0.15 ***	−0.10 ***	0.16 ***	0.60 ***	0.68 ***	-	-	-	-	-	-	-	-	-	-	-	-	-
9	5.18	0.90	−0.23 ***	−0.24 ***	−0.20 ***	−0.15 ***	0.14 ***	0.64 ***	0.64 ***	0.65 ***	-	-	-	-	-	-	-	-	-	-	-	-
10	4.85	1.03	−0.28 ***	−0.30 ***	−0.24 ***	−0.18 ***	0.14 ***	0.57 ***	0.64 ***	0.70 ***	0.60 ***	-	-	-	-	-	-	-	-	-	-	-
11	5.25	0.85	−0.24 ***	−0.26 ***	−0.21 ***	−0.14 ***	0.16 ***	0.59 ***	0.72 ***	0.73 ***	0.66 ***	0.75 ***	-	-	-	-	-	-	-	-	-	-
12	5.32	0.76	−0.22 ***	−0.23 ***	−0.19 ***	−0.13 ***	0.14 ***	0.54 ***	0.63 ***	0.65 ***	0.60 ***	0.65 ***	0.71 ***	-	-	-	-	-	-	-	-	-
13	5.06	0.84	−0.19 ***	−0.20 ***	−0.15 ***	−0.11 ***	0.14 ***	0.54 ***	0.60 ***	0.65 ***	0.60 ***	0.66 ***	0.69 ***	0.69 ***	-	-	-	-	-	-	-	-
14	5.25	0.83	−0.22 ***	−0.23 ***	−0.21 ***	−0.12 ***	0.16 ***	0.53 ***	0.63 ***	0.66 ***	0.58 ***	0.63 ***	0.73 ***	0.71 ***	0.68 ***	-	-	-	-	-	-	-
15	5.21	0.96	−0.16 ***	−0.17 ***	−0.16 ***	−0.07 ***	0.12 ***	0.45 ***	0.53 ***	0.53 ***	0.51 ***	0.53 ***	0.60 ***	0.59 ***	0.55 ***	0.69 ***	-	-	-	-	-	-
16	4.93	0.96	−0.27 ***	−0.28 ***	−0.24 ***	−0.17 ***	0.17 ***	0.54 ***	0.62 ***	0.66 ***	0.60 ***	0.68 ***	0.69 ***	0.66 ***	0.66 ***	0.73 ***	0.62 ***	-	-	-	-	-
17	5.23	0.98	−0.24 ***	−0.24 ***	−0.22 ***	−0.13 ***	0.16 ***	0.52 ***	0.63 ***	0.62 ***	0.55 ***	0.59 ***	0.68 ***	0.61 ***	0.58 ***	0.67 ***	0.54 ***	0.70 ***	-	-	-	-
18	5.23	0.90	−0.19 ***	−0.21 ***	−0.16 ***	−0.09 ***	0.14 ***	0.59 ***	0.62 ***	0.63 ***	0.64 ***	0.62 ***	0.68 ***	0.62 ***	0.66 ***	0.64 ***	0.55 ***	0.64 ***	0.65 ***	-	-	-
19	5.43	0.73	−0.14 ***	−0.14 ***	−0.13 ***	−0.08 ***	0.13 ***	0.52 ***	0.57 ***	0.58 ***	0.59 ***	0.54 ***	0.62 ***	0.58 ***	0.62 ***	0.55 ***	0.51 ***	0.53 ***	0.57 ***	0.70 ***	-	-
20	5.91	1.20	−0.56 ***	−0.56 ***	−0.49 ***	−0.37 ***	0.17 ***	0.49 ***	0.52 ***	0.45 ***	0.43 ***	0.55 ***	0.50 ***	0.43 ***	0.44 ***	0.47 ***	0.39 ***	0.57 ***	0.52 ***	0.47 ***	0.35 ***	-
21	5.24	0.70	−0.32 ***	−0.34 ***	−0.28 ***	−0.19 ***	0.19 ***	0.74 ***	0.82 ***	0.82 ***	0.78 ***	0.82 ***	0.87 ***	0.81 ***	0.81 ***	0.83 ***	0.73 ***	0.84 ***	0.80 ***	0.81 ***	0.73 ***	0.66 ***

Note. V. Variable; M. Mean; SD. Standard deviation. 1. Overall suicidal behavior; 2. Suicidal ideation; 3. Suicide plan; 4. Suicide attempt; 5. Parental marital satisfaction; 6. Bonding; 7. Resilience; 8. Social competence; 9. Recognition for positive behavior; 10. Emotional competence; 11. Cognitive competence; 12. Behavioral competence; 13. Moral competence; 14. Self-determination; 15. Self-efficacy; 16. Clear and positive identity; 17. Beliefs in the future; 18. Prosocial involvement; 19. Prosocial norms; 20. Spirituality; 21. Total positive youth development. *** *p* < 0.001.

**Table 5:** Results of the mediating analysis (N = 3665).

Effect	Point Estimate	S.E.	*p*	Bias-Corrected 95% CI	Percentage of Total Effect (%)
Total effect	−0.11	0.02	<0.001	[−0.15, −0.07]	
Direct effect	−0.05	0.01	<0.001	[−0.09, −0.01]	43.4
Indirect effect	−0.06	0.01	<0.001	[−0.08, −0.04]	56.6

## Data Availability

The raw data supporting the conclusions of this article will be made available by the authors upon request.

## Supplementary Materials

The following supporting information can be downloaded at: https://www.mdpi.com/article/10.3390/ijerph23040468/s1, Table S1: Regression coefficients of hierarchical multiple regression (N = 3665).
