# Supplementary material for: Parental Marital Satisfaction and Suicidal Behavior in Preadolescents and Adolescents: The Mediating Role of Positive Youth Development Attributes
Source: Int J Environ Res Public Health. 2026 Apr 7;23(4):468. doi: 10.3390/ijerph23040468 (PMC13117011; doi:10.3390/ijerph23040468)
Supplement: Supplementary file 1 [file ijerph-23-00468-s001.zip › ijerph-4208453-supplementary.pdf]

Supplementary Table S1. Regression coefficients of hierarchical multiple regression (N = 3,665)

Model	Predictors	Dependent variable																			
		Overall suicidal behavior					Suicide ideation					Suicide plan					Suicide attempt				
1		R	R^2	ΔR^2	F		R	R^2	ΔR^2	F		R	R^2	ΔR^2	F		R	R^2	ΔR^2	F	
		0.085	0.007	0.007**	2.175*		0.097	0.009	0.009**	2.842**		0.079	0.006	0.006*	1.866*		0.048	0.002	0.002	0.700	
		B	S.E.	β	t	VIF	*B*	*S.E.*	*β*	*t*	VIF	*B*	*S.E.*	*β*	*t*	VIF	*B*	*S.E.*	*β*	*t*	VIF
	Age	0.022	0.013	0.084	1.661	9.219	0.031	0.019	0.081	1.608	9.221	0.026	0.014	0.091	1.813	9.221	0.012	0.013	0.046	0.918	9.212
	Male	-0.016	0.015	-0.018	-1.104	1.004	-0.021	0.021	-0.016	-0.989	1.004	-0.018	0.016	-0.020	-1.179	1.004	-0.007	0.014	-0.009	-0.521	1.004
	Female (Ref.)																				
	Grade 4	0.106	0.078	0.075	1.359	9.160	0.122	0.112	0.060	1.084	9.210	0.129	0.084	0.085	1.534	9.210	0.089	0.074	0.067	1.199	9.153
	Grade 5	0.125	0.060	0.124	2.075*	9.879	0.143	0.086	0.098	1.656	9.872	0.149	0.065	0.137	2.306*	9.872	0.089	0.057	0.093	1.563	9.879
	Grade 6	0.095	0.049	0.094	1.928	8.597	0.109	0.071	0.075	1.545	8.596	0.113	0.053	0.104	2.144*	8.596	0.067	0.047	0.070	1.437	8.597
	Grade 7	0.005	0.043	0.004	0.126	3.567	-0.026	0.062	-0.013	-0.429	3.566	0.030	0.046	0.021	0.654	3.566	0.017	0.041	0.013	0.414	3.566
	Grade 8	0.036	0.033	0.032	1.106	3.076	0.029	0.047	0.018	0.609	3.077	0.034	0.035	0.028	0.958	3.077	0.048	0.031	0.045	1.534	3.076
	Grade 9 (Ref.)																				
	Only child	0.020	0.016	0.021	1.251	1.037	0.037	0.023	0.028	1.657	1.037	0.019	0.017	0.019	1.099	1.037	0.005	0.015	0.005	0.320	1.037
	Non-only-child (Ref.)																				
	Urban	-0.006	0.017	-0.006	-0.359	1.140	-0.013	0.024	-0.009	-0.530	1.140	-0.004	0.018	-0.004	-0.205	1.140	0.001	0.016	0.001	0.064	1.140
	Rural(Ref.)																				
	Middle school	-0.042	0.027	-0.046	-1.513	3.416	-0.093	0.040	-0.072	-2.345*	3.410	-0.027	0.030	-0.028	-0.927	3.410	-0.010	0.026	-0.012	-0.384	3.417
	High school	-0.061	0.029	-0.065	-2.126*	3.370	-0.102	0.041	-0.075	-2.478*	3.364	-0.061	0.031	-0.061	-1.999*	3.364	-0.023	0.027	-0.026	-0.839	3.372
	Bachelor's degree or above	-0.084	0.031	-0.077	-2.713**	2.955	-0.163	0.044	-0.105	-3.682***	2.951	-0.067	0.033	-0.058	-2.025*	2.951	-0.027	0.029	-0.026	-0.909	2.957
	Primary school (Ref.)																				
2		R	R^2	ΔR^2	F		R	R^2	ΔR^2	F		R	R^2	ΔR^2	F		R	R^2	ΔR^2	F	
		0.121	0.015	0.008***	4.126***		0.129	0.017	0.008***	4.696***		0.109	0.012	0.006***	3.328***		0.075	0.006	0.004**	1.567*	
		B	*S.E.*	*β*	*t*	VIF	*B*	*S.E.*	*β*	*t*	VIF	*B*	*S.E.*	*β*	*t*	VIF	*B*	*S.E.*	*β*	*t*	VIF
	Age	0.020	0.013	0.077	1.524	9.226	0.028	0.019	0.074	1.475	9.227	0.024	0.014	0.085	1.694	9.227	0.010	0.013	0.042	0.827	9.218
	Male	-0.014	0.014	-0.016	-0.941	1.005	-0.017	0.021	-0.014	-0.826	1.005	-0.016	0.016	-0.017	-1.036	1.005	-0.006	0.014	-0.007	-0.411	1.005
	Female (Ref.)																				
	Grade 4	0.097	0.078	0.069	1.243	9.166	0.109	0.112	0.054	0.971	9.216	0.120	0.084	0.079	1.434	9.216	0.083	0.074	0.062	1.123	9.159
	Grade 5	0.118	0.060	0.117	1.970*	9.885	0.134	0.086	0.092	1.553	9.878	0.143	0.065	0.132	2.215*	9.878	0.085	0.057	0.089	1.492	9.885
	Grade 6	0.091	0.049	0.090	1.860	8.599	0.104	0.070	0.072	1.477	8.598	0.110	0.053	0.101	2.084*	8.598	0.065	0.047	0.068	1.390	8.598
	Grade 7	0.007	0.043	0.005	0.153	3.567	-0.025	0.061	-0.013	-0.403	3.566	0.031	0.046	0.021	0.679	3.566	0.018	0.041	0.014	0.432	3.566
	Grade 8	0.035	0.032	0.031	1.063	3.077	0.026	0.047	0.016	0.564	3.077	0.032	0.035	0.027	0.919	3.077	0.047	0.031	0.044	1.505	3.077
	Grade 9 (Ref.)																				
	Only child	0.010	0.016	0.010	0.617	1.053	0.023	0.023	0.017	1.030	1.053	0.009	0.017	0.009	0.546	1.053	-0.001	0.015	-0.002	-0.097	1.053
	Non-only-child (Ref.)																				
	Urban	-0.007	0.017	-0.008	-0.432	1.140	-0.014	0.024	-0.011	-0.601	1.140	-0.005	0.018	-0.005	-0.268	1.140	0.000	0.016	0.000	0.017	1.140
	Rural(Ref.)																				
	Middle school	-0.036	0.027	-0.040	-1.322	3.421	-0.085	0.039	-0.066	-2.156*	3.415	-0.022	0.030	-0.023	-0.758	3.415	-0.007	0.026	-0.008	-0.254	3.422

	High school	-0.051	0.028	-0.055	-1.798	3.384	-0.088	0.041	-0.065	-2.151*	3.378	-0.052	0.031	-0.052	-1.711	3.378	-0.017	0.027	-0.019	-0.617	3.385
	Bachelor's degree or above	-0.075	0.031	-0.069	-2.431*	2.964	-0.150	0.044	-0.097	-3.403**	2.960	-0.059	0.033	-0.051	-1.776	2.960	-0.021	0.029	-0.020	-0.718	2.966
	Primary school (Ref.)																				
	Parental marital satisfaction	-0.028	0.005	-0.087	-5.230***	1.022	-0.040	0.008	-0.086	-5.167***	1.022	-0.026	0.006	-0.076	-4.556***	1.022	-0.018	0.005	-0.058	-3.457**	1.022
		R	R^2	ΔR^2	F		R	R^2	ΔR^2	F		R	R^2	ΔR^2	F		R	R^2	ΔR^2	F	
		0.332	0.111	0.096***	31.924***		0.345	0.119	0.102***	34.712***		0.294	0.087	0.075***	24.345***		0.203	0.042	0.036***	11.108***	
		B	*S.E.*	*β*	*t*	VIF	*B*	*S.E.*	*β*	*t*	VIF	*B*	*S.E.*	*β*	*t*	VIF	*B*	*S.E.*	*β*	*t*	VIF
	Age	0.016	0.013	0.061	1.272	9.228	0.022	0.018	0.057	1.199	9.230	0.020	0.014	0.071	1.478	9.230	0.008	0.012	0.031	0.633	9.221
	Male	-0.019	0.014	-0.022	-1.375	1.006	-0.026	0.020	-0.020	-1.295	1.006	-0.021	0.015	-0.022	-1.409	1.006	-0.009	0.014	-0.011	-0.662	1.006
	Female (Ref.)																				
	Grade 4	0.129	0.074	0.091	1.740	9.171	0.152	0.106	0.075	1.433	9.220	0.151	0.081	0.100	1.871	9.220	0.100	0.073	0.075	1.369	9.164
	Grade 5	0.154	0.057	0.153	2.704**	9.898	0.186	0.082	0.128	2.281*	9.890	0.177	0.062	0.163	2.856*	9.890	0.105	0.056	0.110	1.877	9.898
	Grade 6	0.119	0.047	0.118	2.554*	8.607	0.145	0.067	0.100	2.171*	8.606	0.137	0.051	0.126	2.694*	8.606	0.081	0.046	0.084	1.755	8.607
	Grade 7	0.064	0.041	0.047	1.573	3.586	0.060	0.058	0.030	1.027	3.584	0.086	0.044	0.058	1.937	3.584	0.050	0.040	0.039	1.260	3.585
	Grade 8	0.059	0.031	0.053	1.909	3.082	0.062	0.044	0.039	1.409	3.082	0.055	0.034	0.046	1.646	3.082	0.061	0.030	0.057	1.991	3.082
	Grade 9 (Ref.)																				
3	Only child	0.018	0.015	0.019	1.190	1.054	0.035	0.021	0.026	1.636	1.053	0.017	0.016	0.017	1.035	1.053	0.003	0.015	0.004	0.215	1.054
	Non-only-child (Ref.)																				
	Urban	-0.005	0.016	-0.005	-0.298	1.140	-0.011	0.022	-0.008	-0.504	1.140	-0.002	0.017	-0.002	-0.138	1.140	0.001	0.015	0.002	0.093	1.140
	Rural(Ref.)																				
	Middle school	-0.012	0.026	-0.013	-0.458	3.429	-0.047	0.037	-0.036	-1.259	3.423	0.001	0.028	0.001	0.023	3.423	0.008	0.026	0.010	0.319	3.430
	High school	-0.019	0.027	-0.020	-0.703	3.396	-0.039	0.039	-0.029	-0.996	3.391	-0.022	0.030	-0.022	-0.745	3.391	0.002	0.027	0.003	0.093	3.398
	Bachelor's degree or above	-0.018	0.029	-0.017	-0.617	2.993	-0.064	0.042	-0.041	-1.521	2.990	-0.005	0.032	-0.004	-0.153	2.990	0.013	0.029	0.012	0.434	2.995
	Primary school (Ref.)																				
	Parental marital satisfaction	-0.009	0.005	-0.028	-1.718	1.059	-0.011	0.007	-0.025	-1.543	1.059	-0.008	0.006	-0.024	-1.441	1.059	-0.007	0.005	-0.022	-1.293	1.059
	PYD attributes	-0.199	0.010	-0.321	-19.686***	1.077	-0.296	0.014	-0.332	-20.442***	1.077	-0.189	0.011	-0.283	-17.148***	1.077	-0.116	0.010	-0.196	-11.592***	1.077

Note. *$p < 0.05$; **$p < 0.01$; ***$p < 0.001$.
